# Following the Principles of the Universe: Lessons from Plants on Individual and Communal Thriving

**DOI:** 10.1093/icb/icad117

**Published:** 2023-08-21

**Authors:** Beronda L Montgomery

**Affiliations:** Department of Biology, Grinnell College, 1121 Park Street, Grinnell, IA 50112, USA

## Abstract

The means by which plants and other organisms exist in and respond to dynamic environments to support their thriving as individuals and in communities provide lessons for humans on sustainable and resilient thriving. First examined in my book, *Lessons from Plants* (Harvard University Press, 2021), I explore herein the following question: “How can plants teach us to be better humans?” I consider how insights gathered from plant physiology, phenotypic plasticity, and other plant growth phenomena can help us improve our lives and our society, with a focus on highlighting academic and scientific environments. Genetically identical plants can have very different appearances, metabolisms, and behaviors if the external environments in which they are growing differ in light or nutrient availability, among other environmental differences. Plants are even capable of transformative behaviors that enable them to maximize their chances of survival in dynamic and sometimes unfriendly environments, while also transforming the environment in which they exist in the process. Highlighting examples from research on, for instance, plants’ responses to light and nutrient cues, I focus on insights for humans derived from lessons from plants. These lessons focus on how plants achieve their own purposes by following common principles of the universe on thriving and resilience as individuals and in communities.

## Introduction

The success and thriving of biological organisms are determined by the impacts of the environment on a specific genotype, which can result in distinct phenotypes or phenotypic plasticity. This is true, to varying degrees, for organisms across the biological spectrum. Phenotypic plasticity occurs in both animals and plants, which exhibit distinctions in mobility and longevity (Borges 2008). Both plants and animals exhibit a range of plasticity responses from rapid responses, for example, behaviors such as moving, or slower responses that include changes in morphology, physiology, or development (Borges 2008). One distinction is that organogenesis occurs throughout the plant life cycle, while occurring mostly during embryogenesis for animals. The modular nature of plant growth relative to animals increases the extent of morphogenic flexibility in plants (Schlichting 1986). Additionally, due to the relative immobility of plants, plasticity may have greater importance for these organisms to adapt in dynamic environments (Borges 2008). In organisms in which phenotypic plasticity occurs, the plasticity phenomenon is often adaptive (Lande 2009; Fusco and Minelli 2010). Plastic responses must be appropriate and/or increase fitness to be viewed as adaptive (Schlichting 1986).

Distinct organisms such as plants and animals that exhibit phenotypic plasticity appear to use similar mechanisms to achieve it, suggesting commonalities or common principles that govern plasticity in biological organisms (Borges 2005, 2008). While we understand that the critical interface between plants and external environmental factors determines ultimate phenotypes, parallels to or lessons about the importance of the individual-environment interface for influencing professional or social outcomes for humans individually and in community are often overlooked (Montgomery 2020c, 2021a, 2021b).

Herein, I reflect on the importance of the presence of critical factors and relationships with other organisms in the environment, as well as the ability of humans to perceive, access, and respond to them, in promoting the success of individuals and communities. I highlight the inspiration that we can draw from reflecting on lessons about plant phenotypic plasticity, including adaptive responses and the development of resilience, as well as symbiosis in which plants are involved, as evidence of efficacious means for promoting the resilience and success of individuals and communities in STEM and academic environments.

## Light and nutrients impact phenotypic plasticity in plants

Light is a signal that strongly influences aspects of plant phenotypic plasticity (Borges 2008). Photomorphogenesis, or the impact of light quality, color, and light intensity on phenotypic plasticity, has been widely studied in plants (Sultan 2000; Gratani 2014). Light is indeed critical for plants, which use light energy during the process of photosynthesis to convert inorganic carbon and water into carbohydrates that serve to support their growth and thriving. Although light is critical, plants exhibit limitations in growth if light levels are below optimal; nevertheless, they can also exhibit stress and growth limitations if their exposure to light exceeds that which can be productively used to drive photosynthesis, resulting in photooxidative stress and potential cellular damage or death. Additionally, abrupt shifts between light conditions, for example, transitions from growth in shade to full sun or vice versa, which are common in natural plant canopies, can result in the induction of plasticity or acclimation responses (Ruban 2009; Ruban 2015; Slattery et al. 2018; Long et al. 2022). Thus, success for plants in dynamic conditions can range from growth when an environment is stable and light is optimal to the promotion of resilience and acclimation when transitions occur. Because of the dynamicity of the light environments in which plants exist, these organisms exhibit finely tuned acclimation responses to optimize growth and development.

Nutrient availability, which is impacted by nutrient levels and accessibility in the soil, also affects phenotypic plasticity in plants. Nutrient levels can influence different aspects of plant growth, including root morphology (Hermans et al. 2006; Song et al. 2019) and plant greening (Tränkner et al. 2018; Nam et al. 2021). Similar to the impact of dynamic light, in highly variable nutrient conditions, plants may expend energy for acclimation or resilience, or in relatively stable conditions, energy may be used primarily for growth and development (e.g., Gruber et al. 2013; Sakuraba 2022). The acclimation of plants to the available nutrients maximizes their survival, thriving, and reproduction.

## Plants as responsive beings

**Fig. 1:**
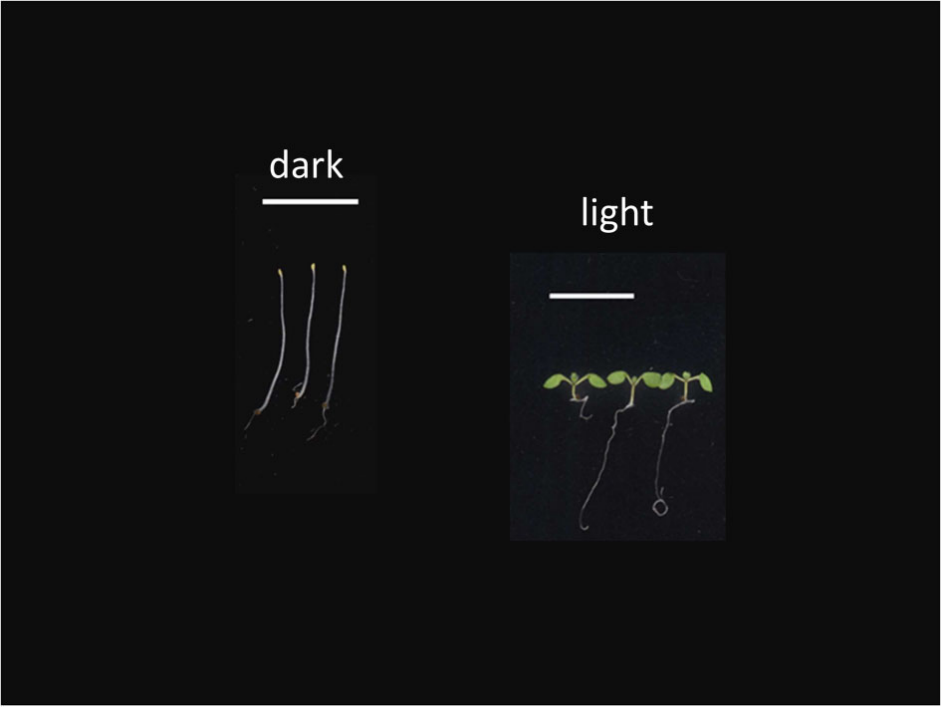
Plants grown in the dark (left) have an appearance that is very distinct from plants grown in the light (right). The depicted seedlings are genetically identical and, apart from the difference of dark versus light, are grown in otherwise identical conditions for 7 days. The ability of organisms to adapt their form and function to distinct conditions, such as light availability, is known as developmental plasticity (a form of phenotypic plasticity) and is linked to the strategic use of the available energy to optimize fitness and survival. The plant shown is *Arabidopsis thaliana*.

Photomorphogenesis is a long-studied form of phenotypic plasticity in plants as well as in photosynthetic microbes such as cyanobacteria that occurs in response to dynamic light environments (Su et al. 2021; Montgomery 2022). There are vast differences in the appearance and physiology of identical seeds germinated in light versus dark that typify photomorphogenic versus skotomorphogenic growth of seedlings, respectively (Fig. 1). These distinctions in the phenotypes of dark- vs. light-grown seedlings demonstrate the responsiveness of plants to external light cues (Fig. 1). Plants grown in the light exhibit short stems and expanded green leaves, as well as a developed root system. Etiolated plants grown in darkness have elongated stems typical of plants searching for light, i.e., unexpanded embryonic leaves or cotyledons, and root systems that are not fully developed. The powerful visible differences observed for dark-grown versus light-grown seedlings serve as an impactful example that equal aptitude—in this case, identical genetic make-up—results in vastly different outcomes depending on the external environment. Discussed here for plants, this is also true for many biological organisms that respond to external environmental cues. This illustration demonstrates behavioral acclimation driven by the energy available. Notedly, the impact of light on growth at the seedling stage can impact positively or negatively aspects of growth, development, and productivity at the adult stage of plants (Hanley and May 2006; Poorter 2007; Muscarella et al. 2013).

As responsive beings, organisms, including plants, expend energy to engage in behaviors and growth or in tuning their developmental patterns to match the external environment. Additionally, plants that have adapted to one environment and transition to another for which their growth and development are not optimized often expend energy to increase survival or resilience (Ruban 2009). This judicious use of energy promotes thriving based on the environment and contrasts with organisms’ spending energy on behaviors or growth patterns that are not linked to survival or thriving. I have previously argued that this represents a powerful lesson for humans in that equal or near-equal aptitude can result in vastly different outcomes depending on environmental access to factors critical for thriving (Montgomery 2021a). This observation is true across multiple contexts, including familial, communal, educational, or professional.

In addition to the phenotypic variability that can be induced due to vastly different external environments, individuals of the same species grown in the same environment can exhibit distinct phenotypes due to inter-individual variability in gene expression, recently investigated for the plant *Arabidopsis thaliana* (Cortijo et al. 2019). Uncovering the high degree of variability in gene expression that can exist between similar plants in an identical environment involved using advanced molecular techniques to determine and compare changes in gene expression between single plant seedlings. Highly variable genes in *A. thaliana* were preferentially enriched for genes that function to prime organisms to be responsive to the environment or stresses (Cortijo et al. 2019). This observed phenomenon results in phenotypic plasticity, which demonstrates that an identical environment can also impact two very similar individuals quite differently. Drawing parallels to humans, we may need to employ advanced approaches to understand what internal dynamics contribute to the distinct observable outcomes of individuals of similar aptitude in an identical context.

When we see differences in human outcomes, even in environments such as science or broader higher education in which we select for a narrow range or relatively similar aptitude, we often attribute success and thriving (or, alternatively, the lack thereof) to differences in individuals’ agency and intrinsic abilities rather than differences in access to or stewardship over external critical factors (Montgomery 2020b, 2020c, 2021a, 2022). I have posited that our potential and morale as individuals and our reciprocal thriving in communities would be vastly improved if we understood ourselves both individually and in communities through a lens of expecting individual growth and critically assessing the impact of the individual-environment interface or supporting system (Montgomery 2021a). Growth-based perspectives and engagement are promoted by us understanding and accessing our critical factors in the environments in which we exist, as well as engaging and supporting each other through environmental stewardship or tending of external environments in support of the thriving of individuals existing therein (Montgomery 2020c). Furthermore, we need to understand broader definitions and models of success that would be expected based on the reality of inter-individual variability rather than a narrow focus on individuals demonstrating identical metrics or measures of success (Montgomery 2020c, 2021a).

## Equality vs. equity: prior experience impacts current and future capacity for thriving and success

In response to growth in distinct environments, plants that have identical growth aptitude can depart in growth patterns and productivity, resulting in them exhibiting distinct specific needs based on their prior experience(s). For example, when comparing genetically identical seedlings grown in darkness to those grown in the presence of the critical factor of light (see Fig. 1), the resources needed to support further thriving and growth differ depending on the prior environmental conditions to which the seedlings were exposed (Gommers and Monte 2018). Plants initially grown in light would simply continue to grow and thrive if maintained in an environment similar or identical to that in which they had already been acclimated. Yet, dark-grown or etiolated seedlings transitioned to optimal light limit further expansion of the hypocotyl and instead promote the development of leaves and greening typical of de-etiolation (Armarego-Marriott et al. 2020). By comparison, dark-grown seedlings transitioned to bright light keep cotyledons closed rather than expanding them to protect the seedlings from photooxidative stress that is associated with excess light (Gommers and Monte 2018).

Based on the equivalence and associated outcomes of exhibiting differences in accrued “capital” (Yosso 2005), dark-grown plants compared to light-grown plants would exhibit differences in photosynthetic productivity if they were subsequently grown in identical light environments, as plants transitioned from dark to light exhibit a lag in attaining maximal levels of photosynthetic efficiency (Pipitone et al. 2021). Thus, if leaf production and photosynthetic efficiency alone are measures of success, dark-grown seedlings transitioned to bright light would not be categorized as successful. However, if exhibiting the appropriate response to a transition to bright light of initiating a protective response were recognized as success, then these seedlings would indeed exhibit success.

Transitioning dark-grown and light-grown seedlings directly to high light would be the equivalent of attempting “equality” with individuals of similar initial aptitude who have accrued differences in potential due to divergent paths—one individual having spent their life to date in an environment lacking resources critical for their thriving, while another has had access to critical factors from the first days of its existence. This also does not account for a need for a different measure of success, that is, appropriate initiation of a protective response, for seedlings transitioned from dark to suboptimal light conditions. Thus, if resilience is also a measure of success rather than merely maximizing photosynthetic productivity, dark-grown plants could perform well relative to light-grown plants. The recognition of the distinct needs that were brought about due to differences in the external environments and taking steps to “reactivate” potential in a plant that had been deprived of access to critical factors needed for its thriving, or ensuring a range of measures for success, would be equitable. Such approaches can be infrequently used in scientific and academic environments, where measures of productivity and metrics of success can be extremely narrowly defined (Whittaker and Montgomery 2022).

In human communities, we often default to providing all individuals with equal—rather than equitable—access to resources without adequately assessing their access to, or deprivation of, critical factors at prior points. Furthermore, we do not always assess and account for distinctions in accrued capital and other factors such as resilience or the induction of energetically costly protective mechanisms that may result in different timing of progress toward success and distinct success pathways and outcomes. We often have singular or narrow measures of success that do not account for diverse histories nor acknowledge that the current limited definition and metrics of success are defined by white supremacy (Moreton-Robinson 2019). Indeed, marginalized and minoritized scholars report their scholarly work being undervalued and unrecognized due to epistemic exclusion, including the limited metrics that recognize impactful, “high quality” scholarship and professional success (Settles et al. 2019, 2021). Deficits in the acknowledgment of value are not limited to individuals but extend in plant biology to an overemphasis of the value of models such as *Arabidopsis* ([Bibr bib25][Bibr bib25]; Marks et al. 2023). Equity departs from this celebration of sameness to platform and recognize the valuable contributions and strengths that comes from diversity and difference.

## Plants as interdependent beings

Plants are exquisitely sensitive to the other beings with whom they exist in a community as well as to external environmental factors, such as the aforementioned light and nutrients. In a community with other plants, bacteria, and fungi, plants may collaborate or compete with these organisms. There, the degree of competition or collaboration has been proposed to be tempered or exacerbated by whether the other plants in the community are kin, friends, or foes, although further studies are warranted (Biedrzycki and Bais 2010; Simonsen et al. 2014; Montgomery 2021a).

Classic examples of plants’ collaboration with bacteria and fungi include the formation of nodules and mycorrhizae, respectively. Nodulation occurs when plants interact with nitrogen-fixing bacteria and form a reciprocal relationship in which the bacteria convert atmospheric nitrogen into a form that serves as a type of fertilizer for themselves and the plants with which they are associated (Vance 2001; Wagner 2011). In return, the plants share excess photosynthetically derived fixed carbon with their bacterial symbionts (Vance 2001; Wagner 2011). Mycorrhizae form through the interactions of fungi in the soil with the roots of plants. This symbiotic interaction increases the water and nutrient uptake of plants, including the uptake of phosphate, an important nutrient for plant growth and development (Vance 2001). Mycorrhizae also facilitate the connection of similar individuals, namely plants or trees, in a community through underground interactions (Montgomery 2021a, 2021b). These belowground connections are known as common mycorrhizal networks (Karst et al. 2023). Belowground connections can be negative, neutral, or positive in terms of impact on the interacting partners. While there has been much focus on the positive implications of common mycorrhizal networks, recent meta-studies indicate that further evidence is needed to truly confirm the functional outcomes of these networks (Henriksson et al. 2023; Karst et al. 2023).

The environmental phenomena of plants and microbes collaborating extend beyond the benefits to the two collaborating partners. The interactions between nitrogen-fixing bacteria and plants result in the production of excess fixed nitrogen that is deposited in the soils in which organisms are growing (Stagnari et al. 2017). This deposition results in enriching the environment not only for the symbionts but also for other organisms growing in proximity in the community. Enriched nitrogen levels also can persist in the soil to support future generations of organisms that will inhabit the space, thereby transforming environments in both the short and long term. This phenomenon of transforming the environment is the basis of rotation cropping, in which nitrogen-fixing crops are periodically grown in a field to restore the productivity of soils (Gardner and Drinkwater 2009; Blesh and Drinkwater 2013).

Transformative responses of plants offer impactful lessons for how we as individuals can collaborate to promote reciprocal success. The importance of collaboration and the means for recognizing collective scholarly efforts rather than more commonly recognized individual success models have been recently highlighted (Whittaker and Montgomery 2022). By navigating environments successfully and collaborating with meaningful partners when our individual efforts are insufficient for supporting our thriving, we can seek to exhibit transformative behaviors that enrich communities for our own thriving, the thriving of others in communities, and ultimately to support intergenerational thriving of others who will inhabit the transformed environments.

## Excess resources and “news” shared in support of community and environmental transformation

In addition to the production of excess resources that enrich an environment exemplified by the symbiotic relationship between nitrogen-fixing bacteria and plants, mycorrhizal-mediated sharing purportedly occurs between seedlings and tall trees in a forest, including among distinct species (Klein et al. 2016). Broad implications of the positive reciprocal impacts of mycorrhizal formation and function on productivity are limited by a paucity of studies. Beyond the sharing of resources such as nutrients and carbon-based sugars used to support growth and thriving, plants also have been purported to share “news” in the form of a chemical language that can support resilience and thriving. For example, organic compounds that comprise defense signals or that serve as allelochemicals that function in plants’ responses to stress can be shared between plants through mycorrhizal networks (Gorzelak et al. 2015). Negative interactions can also occur between plants and fungi (Karst et al. 2023).

The potential reciprocal benefits for young and mature plants that are interconnected by fungi in common mycorrhizal networks are many in regard to sharing resources and helpful information. The ability to share growth-promoting resources such as sugars produced during photosynthesis may be critical for establishing seedlings until they are fully photosynthetically competent, as well as potential signals such as plant defense signals that may serve to prime plants to resist potential damage (Montgomery 2021a, 2021b). These benefits of being connected in community, sharing growth-supporting resources, and sharing heuristic knowledge that may serve as protection offer powerful lessons for promoting connections, beneficial communication, and resource sharing in human communities, including scientific and academic communities. Such communal and collaborative practices would depart from common scientific and academic practices that support and reward individual success or competitive models rather than collective or collaborative efforts (Whittaker and Montgomery 2022).

## Promoting growth through ecosystem stewardship

As described earlier, in plants’ responses to light and nutrients, vast differences can be observed for individuals of identical or very similar aptitude in response to dynamic environments and in communities. Understanding the importance of key factors being available to organisms and the need for organisms to have the capacity to perceive and respond to these factors to support appropriate behavioral responses in context suggests a powerful role for us in supporting individual and communal success in our human environments. While humans have a diverse set of critical factors based on prior experiences and accrued capital compared to plants, for which light is a common critical factor, the ability to perceive, respond, and initiate appropriate support networks to promote appropriate outcomes and success are common needs (Montgomery 2020c).

One of the most important roles in moving from recognition and perception of critical factors to initiating appropriate support networks and productive outcomes is for environmental stewards to function as groundskeepers who tend environments in support of individuals, rather than our more common or default engagement as gatekeepers guarding who gains access and the opportunity to thrive in our environments (Montgomery 2020a). In centering one’s work on being a groundskeeper or environmental steward, significant effort is focused on how environmental factors support the thriving of individuals in context. More commonly used gatekeeping practices focus on individual deficits and presume that differences in behavior and function are solely due to intrinsic abilities or lack thereof, identity, origin, or other factors rather than the significant impacts that external factors can impose on organismal form and function (Montgomery 2020a, 2020c). However, recent analyses and persistent practices point to needed disruptions in gatekeeping and active promotion of groundskeeping, including the disruption of persistent biases in assessment and awarding of federal grants (Ginther et al. 2011, 2016; Taffe and Gilpin 2021; Chen et al. 2022) and longstanding biases in support of scholarly research areas outside of norms established and maintained based on spaces that traditionally privilege white and Western researchers and their perspectives (Baffoe et al. 2014; Settles et al. 2019, 2021).

The observed phenotypic differences for genetically identical seedlings grown in darkness compared to light offer a powerful visual demonstration of the impact of the environment on organismal thriving and persistence. Also, these data provide inspiration for the cultivation of environmental stewards to tend environments to support individuals and communities in context, whereas gatekeeping prioritizes “fixing” individuals to demonstrate narrow visions and metrics in status-quo-keeping environments.

Focusing on the impact of interdependence between plants and other organisms and the individual-environment interface on plants stimulate lessons for us that would allow us to focus on which areas can be stewarded and enable us to recognize other critical aspects of environments that must be changed. That is to say that in supporting a diverse range of individuals in human communities, we need to recognize how to identify the range of critical factors that support distinct individuals when differences in prior environmental contexts have led to observable differences in individuals with similar aptitudes, and how this latter recognition must impact our equitable environmental stewardship to support and restore maximal outcomes for the broadest range of individuals possible.

At the same time, we must acknowledge when aspects of our environments represent structural deficits and barriers (i.e., racism, sexism, etc.), including persistent gaps in achievement and graduation for groups underrepresented in scientific and academic environments compared to those from a majority group and a persistent undervaluing of scholarly work from individuals from groups underrepresented in higher education. Likewise, we must recognize and confront how we cannot mentor our way out of the impacts of these structural features, but in so doing, we must also not default to the common practice of depending on the undue grit of the marginalized and minoritized to support their own persistence and thriving (Montgomery 2020a).

We need to identify and fix the structural barriers and deficits (Whittaker and Montgomery 2012; Whittaker et al. 2015). It is equally important to identify bridges and stages that provide access and platforms that promote success selectively for some and replicate them to increase access and opportunity more broadly. To remove barriers and replicate access points requires ecosystem assessment and stewardship (Mondisa et al. 2021).

## Conclusions

The ways in which biological organisms sense and respond to the external environment to tune their physiologies, metabolisms, and behaviors to external cues can be observed across the biological spectrum. Lessons focusing on how plants respond and acclimate to light and nutrients, engage in collaborative relationships with other organisms such as nitrogen-fixing bacteria that enrich the environment, and benefit from the perspectives and tending of groundskeepers rather than gatekeepers are all examples of the principles of the universe for supporting thriving and limiting damage. Humans often opt out of the universe’s principles of sustainability and reciprocity, which leave us at odds with sustainable living and battling issues such as climate change and organismal extinction. We would do well to look to other organisms, such as plants, for inspiration to promote our individual and communal successes in our generation and beyond.

## Data Availability

All data relevant to this study are either included or referenced to within the main manuscript or its supplemental material.
